# Pancytopenia Unraveled: Tracing Rare Cardiobacterium hominis Bacteremia Back to the Gut

**DOI:** 10.7759/cureus.86358

**Published:** 2025-06-19

**Authors:** Annie Kleynerman, Ryan C Day, Iiro Honkanen

**Affiliations:** 1 Department of Medicine, Medical College of Wisconsin, Milwaukee, USA; 2 Department of Anesthesiology, Medical College of Wisconsin, Milwaukee, USA; 3 Department of Medicine, Division of Nephrology, Medical College of Wisconsin, Milwaukee, USA

**Keywords:** bacteremia, blood cultures, bone marrow suppression, cardiobacterium hominis, delayed speciation, endoscopy, infective endocarditis, lymphocytic colitis, pancytopenia, transesophageal echocardiography

## Abstract

*Cardiobacterium hominis* (*C. hominis*) is part of the normal flora of the human oropharynx. Although uncommon, it is a significant cause of endocarditis, particularly in individuals with pre-existing valvular heart disease. The combination of its rarity, prolonged blood culture incubation time, and nonspecific clinical presentation makes *C. hominis* infections particularly challenging to diagnose. We present the first known published case of pancytopenia secondary to *C. hominis* bacteremia in an elderly male, likely incited by a gastrointestinal endoscopic procedure. We discuss the importance of recognizing signs of subclinical bacteremia and maintaining a broad differential for pancytopenia for timely diagnosis and management.

## Introduction

*Cardiobacterium hominis* (*C. hominis*) is a Gram-negative, fastidious bacillus belonging to the HACEK group, which also includes *Haemophilus paraphrophilus*, *Haemophilus parainfluenzae*,* Aggregatibacter actinomycetemcomitans*, *Aggregatibacter aphrophilus*, *Eikenella corrodens*, and *Kingella kingae*. With fewer than 100 cases reported in the English literature, *C. hominis* remains a rare but important cause of bacteremia, frequently complicated by infective endocarditis in 88-95% of cases [1]. All HACEK organisms are known to be associated with infective endocarditis, a condition that carries a high mortality rate ranging from 8% to 40% [2]. Notably, *C. hominis* infections are diagnostically challenging due to their variability in Gram staining, slow growth in culture media, and nonspecific clinical presentation [1]. With the aortic valve predominantly affected, known risk factors include post-surgical valve treatments, congenital valve abnormalities, dental procedures, and upper gastrointestinal endoscopy [3]. Given its rarity, there are gaps in the literature regarding the clinical presentation and prognosis of *C. hominis* infections. Pancytopenia, defined as a reduction in red blood cells, white blood cells, and platelets, may occur as a result of various conditions, including infection, inflammation, and malignancy. Here, we present the diagnosis and treatment of an elderly male patient with the first published case of pancytopenia resulting from *C. hominis* bacteremia, likely due to an endoscopic investigation.

## Case presentation

A 75-year-old male with a past medical history pertinent for aortic stenosis status post-transcatheter bovine aortic valve replacement (TAVR) and lymphocytic colitis presented to the emergency room with symptoms of volume overload and exertional dyspnea, raising concerns of a new heart failure exacerbation.

Two months prior, he was evaluated for a lower gastrointestinal bleed, and a colonoscopy revealed a flare of lymphocytic colitis, for which he was prescribed budesonide. Upon outpatient follow-up, he reported improved, but persistent diarrhea and abdominal cramping, without any further bleeding. At the time, he was found to have a white blood cell (WBC) count of 2.1 x 10^3^/uL, hemoglobin (Hgb) of 9.6 g/dL, and platelet count (PLT) of 87 x 10^3^/uL. Subsequently, the patient was referred to hematology for further workup, which included a bone marrow biopsy.

**Figure 1:**
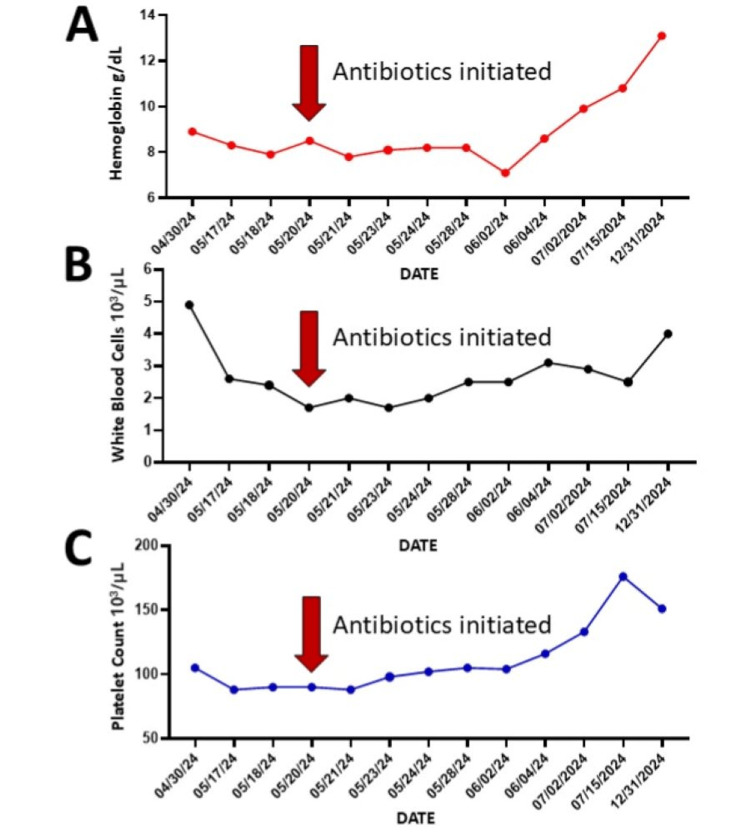
Improvement of pancytopenia after antibiotic treatment Long-term hematologic improvement following a two-week course of antibiotics. Trends in hemoglobin (Hgb, g/dL) (A), white blood cell count (WBC, 10³/µL) (B), and platelet count (PLT, 10³/µL) (C) are shown. Red arrows indicate the initiation of antibiotic treatment on 05/20/2024. Within 14 days, WBC increased from 1.7 to 2.5 (10³/µL) and reached 4.0 (10³/µL) at the six-month follow-up; Hgb rose from 8.5 to 8.6 g/dL within 14 days and 13.1 g/dL at 6 months; PLT count improved from 90 to 116 (10³/µL) within 14 days and 151 (10³/µL) at 6 months.

Upon admission to our team, the patient was treated with diuretics for volume management. A CBC showed a WBC of 2.6 x 10^3^/uL, hemoglobin (Hgb) of 8.3 g/dL, and a platelet count (PLT) of 88 x 10^3^/uL (Figures 1A-1C). The patient remained hemodynamically stable but developed a fever with chills the following morning. The etiology of his pancytopenia and systemic symptoms was unclear. Malignancy or sub-acute infection was highest on the initial differential diagnosis. Further history-taking for infection risk factors revealed only a distant history of a bite from a roadrunner bird.

The infectious workup included an unremarkable urinalysis, a negative chest X-ray, and drawn blood cultures. A CT scan of the abdomen and pelvis was obtained due to suspicions of active colitis; however, imaging did not reveal any abscess, colitis, or other potential infection sources. A transthoracic echocardiogram (TTE) showed normal systolic function with new perivalvular regurgitation of his bioprosthetic aortic valve, raising concern for infectious valvulopathy. Blood samples were cultured slowly, yielding Gram-negative rods in both aerobic and anaerobic bottles at 48 hours. The initial common pathogen panel was negative, so empiric intravenous ceftriaxone was initiated for bacteremia of an unknown source.

**Figure 2:**
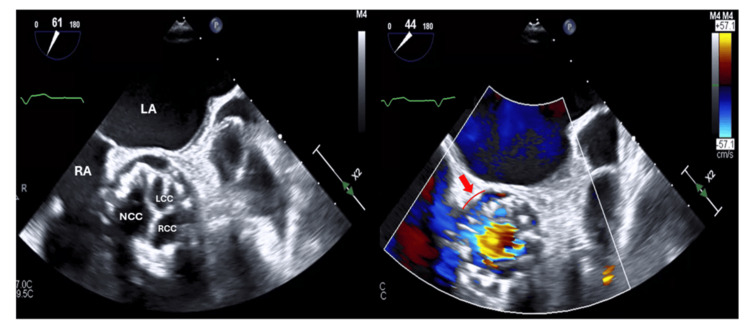
Transesophageal echocardiographic assessment of the bioprosthetic aortic valve with a mild perivalvular leak Transesophageal echocardiogram (ME AV SAX view) in B-mode (left) and with color Doppler (right) of the bioprosthetic valve (BiP-AV) with a demonstration of a mild perivalvular leak (right, red arrow). LA: left atrium, RA: right atrium, NCC: non-coronary cusp BiP-AV, LCC: left coronary cusp BiP-AV, RCC: right coronary cusp BiP-AV

Given the findings of paravalvular leak on TTE in the setting of a prosthetic valve, bacteremia, and subacute pancytopenia, a transesophageal echocardiogram (TEE) was performed (Figure 2). A paravalvular leak was present; however, the TEE was ultimately deemed negative for evidence of endocarditis (Figure 2). Alternatively, bone marrow analysis failed to identify any neoplastic processes to explain his pancytopenia. Final speciation of blood cultures required send-out to an outside facility and took 21 days before pan-susceptible *C. hominis* could be identified.

After the initiation of treatment, the patient had a gradual resolution of his exertional dyspnea, fevers, and chills. All cell lines started to increase (Figures 1A-1C) after the completion of a two-week course of antibiotics. The nadir for his pancytopenia during his presenting hospital admission included a WBC count of 1.7 x 10^3^/uL, Hgb of 7.1 g/dL, and PLT count of 88 x 10^3^/uL (Figures 1A-1C). A month later, his repeat labs revealed an uptrending WBC count of 3.1 x 10^3^/uL, Hgb of 8.6 g/dL, and a PLT count of 116 x 10^3^/uL (Figure 1A-1C). The six-month follow-up confirmed full recovery with labs back to a baseline of WBC 4.0 x 10^3^/uL, Hgb 13.1 g/dL, and PLT 151 x 10^3^/uL (Figures 1A-1C).

## Discussion

*C. hominis* infections are challenging to diagnose due to their rarity, prolonged blood culture duration (2-21 days), and nonspecific clinical symptoms [1]. While *C. hominis* is a normal part of the human oropharyngeal microbiota and rarely causes a disease, it can act as an opportunistic pathogen, particularly affecting heart valves [4]. Endocarditis related to *C. hominis* is often low-grade and insidious [1]. In one case, systemic symptoms appeared nine months before diagnosis [5]. Of 34 cases identified up to 1983, the average time from symptom onset to diagnosis was 169 days [4].

We report, to the best of our knowledge, the first case directly linking *C. hominis* to bone marrow suppression. Furthermore, we discuss the nuances surrounding the diagnosis of *C. hominis* and the workup for suspected infective endocarditis. Our 75-year-old male patient had multiple risk factors, including a history of bovine aortic valve replacement, prior lower gastrointestinal endoscopy, and lymphocytic colitis, which preceded the onset of his symptoms. Initial diagnostic efforts focused on malignancy, heart failure, and infection as the most likely etiologies.

We postulate that *C. hominis *translocated from the gut following a previous endoscopic evaluation or colitis flare, which has been shown to disrupt the intestinal barrier [6]. The bacteremia likely led to a low-grade inflammatory state that contributed to symptoms of fatigue, dyspnea, and peripheral edema. The persistence of *C. hominis* in the blood appears to have resulted in bone marrow suppression, as evidenced by the non-neoplastic pancytopenia that responded to antibacterial treatment. Mechanistically, the cytotoxic LPS-protein complexes released from Gram-negative bacteria like *C. hominis* induce endogenous cytokines that lead to downstream effects like bone marrow suppression, further supporting our hypothesis [7].

Infections arising after gastrointestinal endoscopy can stem from two primary sources: endogenous infections caused by the patient’s own flora and exogenous infections resulting from contamination of the endoscope [6]. Notably, there is only one documented case of *C. hominis* infection following upper gastrointestinal endoscopy, and no cases were reported after colonoscopy procedures [3]. A study by Ahuja et al. reviewed the outcomes of 12,714 patients, identifying 55 cases (0.43%) of bacteremia that developed within 30 days following an endoscopic procedure [6]. The most frequently identified pathogens included *Escherichia coli* (18.18%), *Enterococcus faecalis* (10.91%), *Pseudomonas aeruginosa* (7.27%), and *Klebsiella* species (5.45%) [6]. Among the identified cases, a significant majority (69.09%) were linked to esophagogastroduodenoscopies (EGDs), while 23.64% were associated with colonoscopies [6]. Bacteremia was found to manifest predominantly within 7 to 30 days post-procedure (61.82%) compared to within 7 days (38.18%) [6]. Our patient underwent a colonoscopy 36 days before the detection of pancytopenia and 70 days prior to the hospital admission when bacteremia was diagnosed.

Echocardiography was imperative to further evaluate our patient, especially in the setting of new-onset perivalvular flow. As our patient had a history of a prosthetic aortic valve, seeding of the valve became a prominent concern. *C. hominis*-related endocarditis has an insidious onset, and diagnosis is often delayed by an average of six months up to a year after symptoms develop [5]. Of the 61 reported cases in the literature, more than a quarter involved prosthetic valves [1]. Non-invasive TTE was the initial choice but provided an equivocal assessment of the aortic valve. TEE is nearly twice as sensitive as TTE (86-94% vs. 36-69%) and comparably specific (88-100%) for detecting prosthetic valve vegetations in endocarditis [8]. Following our patient’s negative TEE evaluation, valvulopathy could be excluded, and the antibiotic course appropriately adjusted.

## Conclusions

This case highlights chronic, subclinical *C. hominis* bacteremia as a cause of persistent pancytopenia and constitutional symptoms that remained undiagnosed for several months. We emphasize the importance of including bacteremia in the differential for pancytopenia and nonspecific symptoms, particularly in patients with inflammatory conditions, immunosuppressive therapy, or recent endoscopic procedures. Identifying bacteremia and assessing pathogen predilections in conjunction with patient risk factors can help guide decision-making regarding further cardiac or CT imaging. In this case, prompt blood culture results facilitated timely antibiotic treatment, exclusion of cardiac valve involvement, and a promising recovery.
